# Prognostic Role of the Neutrophil-to-Lymphocyte Ratio in Intracerebral Hemorrhage: A Systematic Review and Meta-Analysis

**DOI:** 10.3389/fnins.2022.825859

**Published:** 2022-03-10

**Authors:** Min Shi, Xiao-feng Li, Ting-bao Zhang, Qing-wen Tang, Mian Peng, Wen-yuan Zhao

**Affiliations:** ^1^Department of Neurosurgery, Zhongnan Hospital of Wuhan University, Wuhan, China; ^2^Department of Anesthesiology, Zhongnan Hospital of Wuhan University, Wuhan, China

**Keywords:** neutrophil-to-lymphocyte ratio, intracerebral hemorrhage, prognostic value, mortality, poor outcome, hematoma expansion, neurological deterioration

## Abstract

The neutrophil-to-lymphocyte ratio (NLR) plays an important role in the progression of intracerebral hemorrhage (ICH). An increasing number of studies have reported that a high NLR is correlated with poor clinical outcomes among patients with ICH. Here, we conducted a systematic review and meta-analysis to evaluate the prognostic value of NLR in the setting of ICH. We performed a comprehensive search of electronic literature databases to identify all relevant studies evaluating the prognostic role of NLR in patients with ICH. Two researchers independently screened the studies and extracted relevant data. We extracted, pooled, and weighted odds ratio (OR) and 95% confidence interval (CI) values using a generic inverse-variance method, and then evaluated the heterogeneity among studies using *Q* test and *I*^2^ statistic. Finally, we selected a total of 26 studies including 7,317 patients for the current study. Overall, our results indicated that a high NLR was significantly associated with a poor outcome (OR, 1.32; 95% CI, 1.19–1.46; *P* < 0.00001), mortality (OR, 1.05; 95% CI, 1.01–1.09; *P* = 0.02), and neurological deterioration (OR, 1.65; 95% CI, 1.08–2.52; *P* = 0.02). We did not observe a significant association between NLR and hematoma expansion (OR, 1.04; 95% CI, 0.99–1.08; *P* = 0.09). Our study indicated that a high NLR is significantly associated with poor clinical outcomes in patients with ICH. As NLR is a simple and easily available biomarker, future studies should focus on exploring its application in the prognostic evaluation of patients with ICH.

## Introduction

Spontaneous intracerebral hemorrhage (ICH), as a common stroke subtype, is one the most devastating neurosurgical diseases, with high mortality and morbidity rates (Feigin et al., 2003; van Asch et al., 2010). ICH is characterized by an instance of sudden bleeding due to ruptured cerebral blood vessels and by the action of blood flowing into the brain parenchyma or cerebral ventricles (Keep et al., 2012). Despite the great advances and developments that have occurred in medical technology, ICH still represents the most serious type of stroke due to a persistent lack of effective therapeutic strategies (Hwang et al., 2011; Cordonnier et al., 2018). It is reported that more than one-third of patients who experience ICH die within one month after ictus, and many of the survivors are severely disabled and show persistent functional impairment (Ferro, 2006; van Asch et al., 2010; Ikram et al., 2012). ICH thus represents a substantial clinical and economic public health burden, especially in low-income countries, and it is critical to identify promising prognostic factors to evaluate the severity and prognosis of patients (Wang W. et al., 2017; Garg and Biller, 2019).

Intracerebral hemorrhage scores, consisting mainly of the Glasgow Coma Scale score, age, ICH volume, intraventricular hemorrhage, and infratentorial origin of ICH, have been extensively validated and widely used to predict poor prognosis among patients with ICH (Hemphill et al., 2001; Nisar et al., 2018). In addition to these risk factors, increasing evidence indicates that an inflammatory response is involved in the development of secondary brain injuries after ICH onset and plays a vital role in the pathophysiological mechanism of ICH (Xi et al., 2006; Wang, 2010; Chen et al., 2015; Wang T. et al., 2018). The neutrophil-to-lymphocyte ratio (NLR), defined as the ratio between the absolute neutrophil count and the absolute lymphocyte count, is an indicator of systemic inflammation that can suggest a patient’s inflammatory status (Wang Y. et al., 2018; Song et al., 2021). Recently, NLR has been widely reported as an independent predictive factor for the prognosis of ICH (Ye et al., 2017; Lattanzi et al., 2019; Liu et al., 2019). In a previous study, Liu et al. (2019) reported that a higher NLR level was significantly correlated with major disability at 90 days and a higher short-term mortality rate. Wang Z. et al. (2019) suggested that NLR can predict hematoma expansion. Finally, Lattanzi et al. (2017) indicated that NLR may be a prognostic factor for neurological deterioration. With the publications of most recent studies, there is still a need to further assess the prognostic value of NLR in terms of poor outcome, mortality, hematoma expansion, and neurological deterioration.

## Methods

We completed the current study according to the Preferred Reporting Items for Systematic Reviews and Meta-Analyses (PRISMA) guidelines (Supplementary File 1).

### Data Sources and Searches

We conducted a comprehensive database search of PubMed, Embase, the Cochrane Library, and the Web of Science, respectively, from database inception until September 2021 to identify all relevant articles. The predefined search strategy was as follows: (“neutrophil-to-lymphocyte ratio” or “neutrophil to lymphocyte ratio” or “neutrophil lymphocyte ratio” or “neutrophil/lymphocyte ratio” or “NLR”) and (“intracerebral hemorrhage” or “intracranial hemorrhage” or “cerebral hemorrhage” or “brain hemorrhage” or “ICH”). The free terms are presented in Supplementary File 2. In addition, we also attempted to manually search all reference lists of the selected studies and related review articles to identify any additional studies that may be appropriate to include.

### Literature Selection and Inclusion Criteria

Two researchers independently completed the literature screening process wherein all potential articles were individually assessed for inclusion in our analysis and any disagreements were solved through discussion between them or involvement of a third researcher. Articles were enrolled if they met the following criteria: (1) included adult patients (≥18 years of age) diagnosed with ICH; (2) reported the NLR values at hospital admission; (3) reported the prognostic value of NLR and clinical outcomes (including poor outcome, mortality, hematoma expansion, and neurological deterioration); (4) provided original data with adjusted odds ratio (OR) and corresponding 95% confidence interval (CI) values in a multivariate analysis for clinical outcomes; and (5) had an observational study design (including prospective and retrospective cohort studies). We excluded articles if they satisfied any of the following criteria: (1) letters, case reports, reviews, or animal studies; (2) only provided univariate analysis data; and (3) did not contain any relevant clinical outcomes.

### Outcome Measures

We included four outcome measures in the current study, including mortality, poor outcome, hematoma expansion, and neurological deterioration. We defined mortality as death from any cause during the follow-up period. We defined a poor outcome by the recording of a Modified Rankin Scale score of more than two points and/or a Glasgow Outcome Scale score of less than four points at any point during follow-up. We defined hematoma expansion by an absolute increase of more than 6 mL or a relative growth of greater than 33% in the hematoma volume using follow-up computed tomography imaging. Finally, we defined neurological deterioration by a National Institutes of Health Stroke Scale score increase of at least four points, a Glasgow Coma Scale decrease of at least two points, or the occurrence of death between the time of admission and 7 days after the hemorrhage.

### Data Extraction and Quality Assessment

Two researchers independently extracted detailed information from individual studies based on a predesigned checklist, including the first author’s name, publication year, country, number of participants, sex ratio, mean or median age of the participants, NLR cut-off value, follow-up period, clinical outcomes (including poor outcome, mortality, hematoma expansion, and neurological deterioration), and corresponding ORs and 95% CI values. ORs and 95% CI values were extracted from multivariable analyses. In addition, we preferentially extracted ORs that represented the association of NLR at admission or at the early phase of admission and outcome measures when available.

We conducted a quality assessment of each included study using the Newcastle–Ottawa Quality Assessment Scale (NOS), which includes eight items, classified into the three domains of selection, comparability, and exposure, respectively. Two researchers independently assessed the quality of each study. The highest NOS score possible was nine points, and we considered studies with NOS scores of at least six points as high-quality studies (Supplementary File 3).

### Statistics Analyses

We pooled the extracted OR and corresponding 95% CI values using the Review Manager version 5.4 analysis software (The Cochrane Collaboration, London, United Kingdom). Forest plots were drawn to display the pooled results. Extracted hazard ratios and 95% CIs were pooled and weighted using a generic inverse-variance method. We used *I*^2^ statistics and Cochran’s *Q* test to assess the heterogeneity across studies. When *I*^2^was greater than?50% or the *P*-value was less than 0.1, we considered the degree of heterogeneity to be substantial and adopted a random-effects model; otherwise, we adopted a fixed-effects model. To further explore the potential source of heterogeneity, we performed a series of subgroup analyses and sensitivity analyses. The subgroup analyses were conducted based on ethnicity, NLR cut-off value, and follow-up period, and the sensitivity analyses were performed by excluding studies one by one. Besides, we further assess the publication bias using a funnel plot.

## Results

### Study Selection and Baseline Characteristic of Included Studies

**FIGURE 1:**
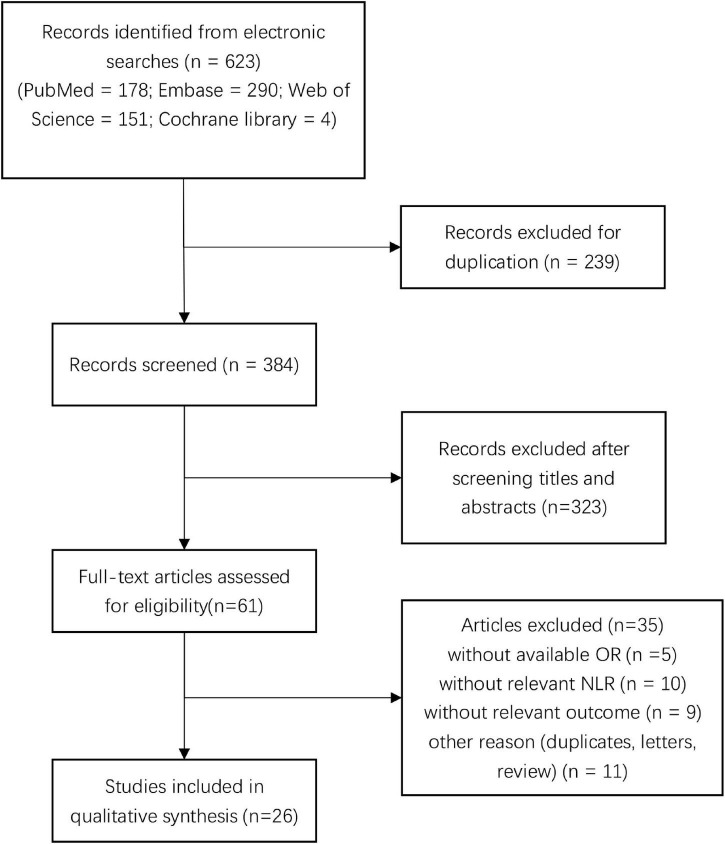
Flow diagram of the study selection process.

We initially screened a total of 623 studies that satisfied our preliminary screening criteria. Figure 1 shows the detailed study-selection process. First, we excluded 239 duplicates, then an additional 323 studies after screening the titles and abstracts; subsequently, 61 studies remained to undergo full-text review. Finally, we included 26 studies with 7,317 patients in the final analysis (Lattanzi et al., 2016, 2017, 2018; Wang et al., 2016; Giede-Jeppe et al., 2017; Seabra et al., 2017; Sun et al., 2017; Tao et al., 2017; Fan et al., 2018; Qi et al., 2018; Wang F. et al., 2018, 2019; Zhang et al., 2018a,b, 2019a,b, 2019c; Guo et al., 2019; Pektezel et al., 2019; Qin et al., 2019; Wang Z. et al., 2019; Chen et al., 2020; Fonseca et al., 2021; Menon et al., 2021; Mohamed et al., 2021; Radu et al., 2021), of which 14 studies reported the prognostic value of NLR for poor outcome, 12 studies reported the prognostic value of NLR for mortality, four studies reported the prognostic value of NLR for hematoma expansion, and three studies reported the prognostic value of NLR for neurological deterioration, respectively.

The baseline characteristics of the included studies are presented in Table 1. All studies were published after 2016, and one was a conference paper, providing limited information. Most studies were conducted in China. Except for two prospective studies, all of the included studies were retrospective investigations. The number of study participants per study ranged from 51 to 855, with a mean age range across all studies of 46.1 to 73 years. The longest follow-up period was 1 year. In all included studies, the NLR was gleaned from peripheral blood samples collected prior to treatment, and the cut-off NLR value ranged from 4.58 to 12.97.

**TABLE 1:** Baseline characteristics of the included studies.

Study	Country	Design	*N* (M/F)	Mean age	Sample time	FUP	Outcome	Cut-off value
Fonseca et al., 2021	Portugal	R	135 (69/66)	73 (64–80)	Admission	90 days	30-day mortality, HE	7.8
Menon et al., 2021	India	R	851 (604/247)	58.09 ± 12.85	Admission	90 days	90-day poor outcome	8.2
Mohamed et al., 2021	Egypt	R	80 (61/19)	57.132 ± 11.37	Admission	7 days	ND	9.1
Radu et al., 2021	Romania	R	201 (111/90)	70 (61–79)	Admission, 3 days	30 days	Hospital mortality	6.3
Chen et al., 2020	China	R	380 (255/125)	58.7 ± 11.4	Admission, postoperative	30 days	30-day mortality	12.97
Guo et al., 2019	China	R	171 (94/77)	46.1 ± 17.2	Admission	90 days	Discharge outcome, 90-day poor outcome	8.25
Pektezel et al., 2019	Turkey	P	383 (223/160)	65 ± 13	Admission, 24th-hour	Discharge	Hospital mortality, HE	NR
Qin et al., 2019	China	R	213 (157/56)	50 (46–55)	Admission	90 days	90-day poor outcome	NR
Wang F. et al., 2018	China	R	275 (207/68)	69 (53, 79)*^a^* 71 (52, 82)*^b^*	Admission	30 days	30-day mortality	NR
Wang Z. et al., 2019	China	R	123 (91/32)	63.01 ± 10.34	Admission	24 h	HE	6.49
Zhang et al., 2019a	China	R	175 (124/51)	60.06 ± 13.01	Admission	30 days	30-day poor outcome	8.20
Zhang et al., 2019b	China	R	107 (72/35)	54.74 ± 12.04	Admission	30 days	30-day poor outcome	7.04
Zhang et al., 2019c	China	R	481 (350/131)	61.09 ± 12.14	Admission	180 days	180-day poor outcome, 180-day mortality	8.69/9.07
Lattanzi et al., 2018	Italy	R	208 (132/76)	66.7 (12.4)	Admission	30 days	30-day poor outcome	NR
Qi et al., 2018	China	R	558 (368/190)	57.6 (28–79)	Admission	90 days	90-day mortality, ND	10.24
Wang T. et al., 2018	China	R	181 (112/69)	65.8 ± 14.3	Next morning	30 days	30-day mortality	7.35
Zhang et al., 2018a	China	R	279 (207//72)	56.59 ± 11.95	Admission	Hospital	HE	14.53
Zhang et al., 2018b	China	R	104 (80/24)	50.40 ± 9.85	Admission	90 days	90-day poor outcome	6.46
Fan et al., 2018	China	R	225 (176/49)	53.20 ± 10.74	Admission	90 days	90-day poor outcome	6.65
Giede-Jeppe et al., 2017	Germany	R	855 (457/398)	72.5 (61–80)*^c^* 71 (62–78)*^d^*	Admission	90 days	90-day poor outcome, hospital/90-day mortality	4.66
Lattanzi et al., 2017	Italy	R	192 (123/69)	66.9 (12.5)	Admission	7 days	ND	NR
Seabra et al., 2017	Portugal	R	51	NR	NR	90 days	90-day poor outcome	NR
Sun et al., 2017	China	P	352 (234/118)	64.2 ± 13.8	Within 24 h	90 days	90-day poor outcome, 90-day mortality	7.85
Tao et al., 2017	China	R	336 (216/120)	58.5 ± 13.0	Admission	90 days	90-day poor outcome, 90-day mortality	6.28/6.62
Lattanzi et al., 2016	Italy	R	177 (63/114)	67.1 ± 12.51	Admission	90 days	90-day poor outcome	4.58
Wang et al., 2016	China	R	224 (141/83)	67.97 ± 13.75	Admission, next morning	30 days	30-day mortality	7.35

*M, male; F, female; FUP, follow-up period; R, retrospective; P, prospective; h, hour; HE, hematoma expansion; ND, neurological deterioration. a, age for survived group; b, age for died group; c, age for NLR≥4.66 group; d, age for NLR<4.66 group.*

### Poor Outcome

**FIGURE 2:**
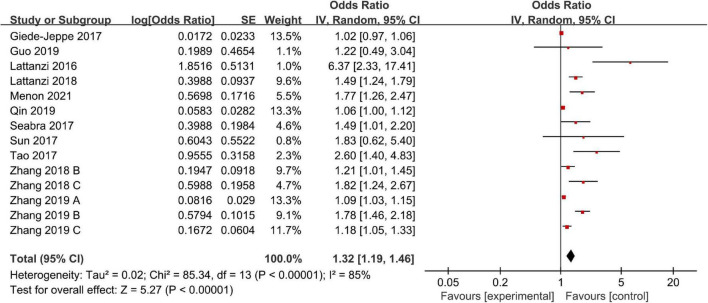
Meta-analysis of the association between NLR and poor outcome.

There were 14 studies including 4,306 patients that reported adjusted ORs for poor outcome. The overall analysis revealed that a high NLR was significantly associated with a poor outcome (OR, 1.32; 95% CI, 1.19–1.46; *P* < 0.00001), and we confirmed statistically significant heterogeneity across all studies (*I*^2^ = 85%; *P* < 0.00001) (Figure 2). Next, we performed a sensitivity analysis by excluding studies one by one, and we found that the meta-analysis results were robust because the ORs did not obviously change after excluding any single study.

We also performed a subgroup analysis based on ethnicity, NLR cut-off value, and follow-up period. The results of the subgroup analysis stratified by ethnicity indicated that a higher NLR was significantly associated with poor outcome in both Asian (OR, 1.32; 95% CI, 1.17–1.49; *P* < 0.00001) and non-Asian (OR, 1.51; 95% CI, 1.04–2.18; *P* = 0.03) regions. According to the NLR cut-off value, we found that there was a significant association between the NLR and poor outcome in the NLR cut-off value of less than 7.5 group (OR, 1.66; 95% CI, 1.21–2.28; *P* = 0.002), and the NLR cut-off value of at least 7.5 group (OR, 1.21; 95% CI, 1.05–1.40; *P* = 0.01). The subgroup analysis indicated that heterogeneity obviously decreased in the higher NLR cut-off value subgroup. It suggests that NLR cut-off value may be the potential source of heterogeneity. Finally, according to the follow-up period, we found that a high NLR was more significantly associated with a poor short-term outcome (<3 months) (OR, 1.41; 95% CI, 1.04–1.91; *P* = 0.03) than a poor relatively long-term outcome (≥3 months) (OR, 1.28; 95% CI, 1.14–1.44; *P* < 0.0001) (Table 2).

**TABLE 2:** Subgroup analysis of the association of NLR with poor outcome and mortality.

Subgroup	Number of studies	*I*^2^ (%)	*P*-value for heterogeneity	Model	OR (95% CI)	*P*-value for statistic
**NLR and poor outcome**						
Total	14	85	<0.00001	Random	1.32 (1.19–1.46)	<0.00001
**Ethnicity**						
Asian	10	82	<0.00001	Random	1.32 (1.17–1.49)	<0.00001
Non-asian	4	90	<0.00001	Random	1.51 (1.04–2.18)	0.03
**NLR cut-off value**						
≥7.5	5	59	0.04	Random	1.21 (1.05–1.40)	0.01
<7.5	6	92	<0.00001	Random	1.66 (1.21–2.28)	0.002
**Follow-up period**						
Short-term (<3 months)	4	90	<0.00001	Random	1.41 (1.04–1.91)	0.03
Long-term (≥3 months)	11	80	<0.00001	Random	1.28 (1.14–1.44)	<0.0001
**NLR and mortality**						
Total	12	73	<0.0001	Random	1.05 (1.01–1.09)	0.02
**Ethnicity**						
Asian	8	80	<0.00001	Random	1.09 (1.01–1.18)	0.02
Non-asian	4	0	0.61	Random	1.03 (1.01–1.05)	0.01
NLR cut-off value						
≥8.0	3	71	0.03	Random	1.07 (1.02–1.12)	0.01
<8.0	7	80	<0.0001	Random	1.07 (0.94–1.23)	0.32
**Follow-up period**						
Short-term (<3 months)	8	22	0.26	Random	1.03 (1.01–1.05)	0.003
Long-term (≥3 months)	5	87	<0.00001	Random	1.12 (1.02–1.23)	0.02

### Mortality

**FIGURE 3:**
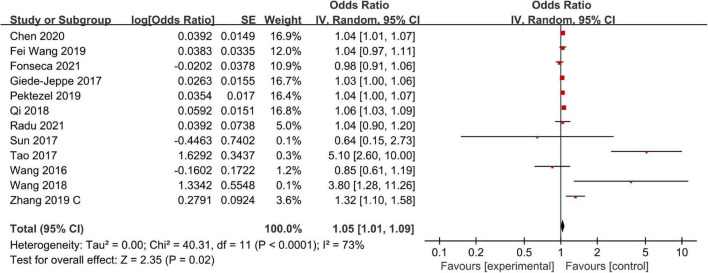
Meta-analysis of the association between NLR and mortality.

There were 12 studies including 4,361 patients that reported adjusted ORs for mortality at the final follow-up. The overall analysis revealed that a high NLR was significantly associated with mortality (OR, 1.05; 95% CI, 1.01–1.09; *P* = 0.02), and statistically significant heterogeneity was confirmed across all studies (*I*^2^ = 73%; *P* < 0.0001) (Figure 3). Next, we performed a series of sensitivity analyses and subgroup analyses to identify potential sources of heterogeneity across studies. The sensitivity analysis was conducted by excluding studies one by one. After removing Tao et al.’s (2017) study, heterogeneity was substantially decreased (*I*^2^ = 47%; *P* = 0.04), which indicated that it may be the potential source of the heterogeneity. However, removing this study did not change the overall pattern of results (OR = 1.04, 95% CI, 1.01–1.07; *P* = 0.002), which indicated that our results were robust.

Separately, we performed subgroup analysis based on ethnicity, NLR cut-off value, and follow-up period. The results of the subgroup analysis stratified by ethnicity indicated that a higher NLR was significantly associated with mortality in both Asian (OR, 1.09; 95% CI, 1.01–1.18; *P* = 0.02) and non-Asian (OR, 1.03; 95% CI, 1.01–1.05; *P* = 0.01) regions. Based on the NLR cut-off value, we found that there was a significant association between NLR and mortality in both the NLR cut-off value of less than 8.0 group (OR, 1.07; 95% CI, 0.94–1.23; *P* = 0.32) and the NLR cut-off value of at least 8.0 group (OR, 1.07; 95% CI, 1.02–1.12; *P* = 0.01). When considering the follow-up period, we found that a high NLR was significantly associated with both short-term mortality (OR, 1.03; 95% CI, 1.01–1.05; *P* = 0.003) and relativity long-term mortality (OR, 1.12; 95% CI, 1.02–1.23; *P* = 0.02) (Table 2).

### Hematoma Expansion

**FIGURE 4:**
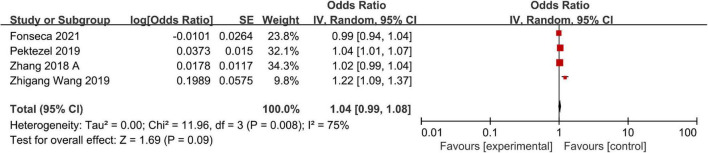
Meta-analysis of the association between NLR and hematoma expansion.

A total of four studies including a total of 920 patients reported adjusted ORs for hematoma expansion. Significant heterogeneity was observed across studies, so we adopted a random-effects model to finish the data synthesis (*I*^2^ = 75%; *P* = 0.008). Ultimately, the pooled results indicated that a high NLR was not significantly associated with hematoma expansion (OR, 1.04; 95% CI, 0.99–1.08; *P* = 0.09) (Figure 4). The sensitivity analysis indicated that the study of Wang Z. et al. (2019) maybe the main source of heterogeneity. After excluding the study, the heterogeneity was obviously decreased (*I*^2^ = 24%; *P* = 0.27). However, removing this study did not change the overall pattern of results (OR = 1.02, 95% CI, 1.00–1.04; *P* = 0.05).

### Neurological Deterioration

**FIGURE 5:**
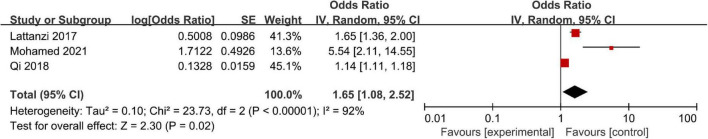
Meta-analysis of the association between NLR and neurological deterioration.

Three studies including a total of 830 patients reported adjusted ORs for neurological deterioration. Significant heterogeneity was observed across these studies, so we adopted a random-effects model to finish the data synthesis (*I*^2^ = 92%; *P* < 0.00001), and the pooled results indicated that a high NLR was associated with neurological deterioration (OR, 1.65; 95% CI, 1.08–2.52; *P* = 0.02) (Figure 5).

### Publication Bias

In the current study, we only tested the publication bias for mortality and poor outcome. Overall, we did not find that obvious publication bias existed for mortality or poor outcome (Supplementary File 4).

## Discussion

A high NLR has been suggested as a prognostic biomarker and is already widely used in patients with cancer and cardiovascular and cerebrovascular diseases (Templeton et al., 2014; Angkananard et al., 2018; Sharma et al., 2021). In the current study, we included 26 studies with a total of 7,317 participants that assessed the prognostic value of NLR in patients with ICH. Fourteen of the included studies evaluated the association between NLR and poor outcome, while 12 studies evaluated the association between NLR and all-cause mortality, four studies evaluated the association between NLR and hematoma expansion, and three studies evaluated the association between NLR and neurological deterioration. Our results indicated that a higher NLR level was significantly associated with all-cause mortality, poor outcome, and neurological deterioration, but we did not observe a significant association between NLR and hematoma expansion.

Neutrophil-to-lymphocyte ratio, considered to be a convenient indicator for systemic inflammation, represents the balance between the systemic inflammatory response and immune response (Maestrini et al., 2015; Malhotra et al., 2018). Increasing studies have indicated NLR may be a reliable predictor of clinical outcome in the setting of ICH (Lattanzi et al., 2019; Tschoe et al., 2020); however, the exact mechanism in this context is not fully understood. Some studies have proposed that NLR may be indicative of inflammation, and a higher NLR level indicates a higher inflammatory level (Chang et al., 2020). In patients with ICH, an inflammation response immediately occurs after the hemorrhage (Slevin et al., 2020; Nóbrega Lima Rodrigues de Morais et al., 2021); following the hemorrhage, hematoma components spread to the brain parenchyma, which can activate microglia to initiate inflammatory signaling. Subsequently, pro-inflammatory cytokines and chemokines are released to favor peripheral inflammatory infiltration, which leads to secondary damage (Lan et al., 2017; Sosa et al., 2018).

Available evidence suggests that circulating neutrophils promptly migrate to the site of hematoma and cerebral injury, and neutrophils are the earliest leukocytes to travel to the hematoma site (Wang and Doré, 2007; Hermann et al., 2018). On the one hand, elevated neutrophils can induce blood–brain barrier injury and exacerbate brain damage by the release of tumor necrosis factor, elastase, matrix metalloproteinase, myeloperoxidase, and reactive oxygen species, inducing neurotoxicity. On the other hand, elevated neutrophils can cause a state of temporary immune suppression, thereby leading to a decrease in peripheral lymphopenia (Römer et al., 2015). In addition, the abrupt release of some amount of catecholamines and steroids can weaken and even fully suppress the immune response in the early stages of hemorrhage, contributing to a decrease in peripheral lymphocytes (Liesz et al., 2013). Lymphocytes are the most important player of adaptive immune responses, and their decrease can enhance the patient’s susceptibility to infection (Wang Y. et al., 2017; Lattanzi et al., 2019). The inflammatory response and immune system are collectively involved in the disease course that results in leukocytosis and lymphocytopenia. The NLR is believed to be a composite index that reflects the balance between the innate (neutrophil) and adaptive (lymphocyte) immune responses. As such, dynamic changes in the NLR can rapidly reflect the possibility of a secondary brain injury and a patient’s vulnerability to posthemorrhage complications (Lattanzi et al., 2019; Kashiwazaki et al., 2021). Therefore, an increase in the NLR value may be a reliable predictor of poor functional outcome, mortality, growth of a hematoma, the risk of developing infection, and early neurological deterioration (Qi et al., 2018; Wang Z. et al., 2019; Fonseca et al., 2021; Menon et al., 2021).

Increasing evidence has indicated that NLR may be a potential prognostic predictor for poor prognosis in patients with ICH (Lattanzi et al., 2019; Liu et al., 2019). However, available results are contradictory. In a previous meta-analysis, Zhang et al. (2017) indicated that there was a correlation between NLR and poor functional outcome, but not the 90-day mortality rate. Ye et al. (2017) found that a high NLR was significantly associated with mortality but not poor functional outcome. Liu et al. (2019) updated a meta-analysis in 2019, and their results indicated that NLR is an independent predictor of major disability at 90 days and short-term mortality among patients with ICH but not of in-hospital mortality or 90-day mortality. Based on these conflicting results, we saw a need to perform an additional meta-analysis. Compared with previous studies, in our investigation, we included more patients and more studies published after 2019. We only included data adjusted by multivariable analysis to weak the degree of contamination by other confounding factors. In addition, we not only evaluated the prognostic value of NLR for mortality and poor outcome but also the prognostic value of NLR on hematoma expansion and neurological deterioration. Therefore, we consider our results to be more reliable and valuable than those of earlier studies.

In the current study, our results indicated that NLR was significantly related to poor outcome and mortality. We further conducted analyses based on different ethnicities, cut-off value, and follow-up period and found that a high NLR is significantly associated with mortality and poor outcome independently of variations in these characteristics. Despite a high NLR had a greater magnitude of association with mortality and poor outcome in certain subgroups, it did not significantly alter the effect of NLR. Substantial heterogeneity was also observed in the current study, and we considered that some factors may explain the origin of such heterogeneity. First, all studies included participants diagnosed with ICH but did not report the initial severity of ictus. Therefore, the initial severity of ictus may have been extremely different between studies. Second, the types and the locations of ICH were not the same. In addition, although most studies provided data at the time of admission, there were still some differences in the timing of sample collection, which can result in heterogeneity because the NLR value can change rapidly at different time points during the disease course. Therefore, we should view these results with caution.

The majority of patients with ICH had a poor prognosis. It is necessary to identify potentially severe patients early, transfer them to the intensive care unit, and initiate effective management on time. Currently, the ICH score is the most commonly used clinical predictive model for identifying ICH patients with unfavorable prognoses. However, the ICH score, just based on clinical information, ignores the predictive value of biological elements from the lab. The findings of our study fill this gap in the field and help with future guidelines addressing the topic of the prognostic value of NLR in ICH. Future studies should focus on investigating the association between NLR and clinical outcomes in specific subgroups, including stratification by subtype of hemorrhage, location of hemorrhage, or severity of ICH.

There were some limitations in this meta-analysis. First, most of the included studies were retrospective investigations, which may have led to inevitable selection and information bias. Second, there was substantial heterogeneity among the included studies. Although we explored the source of heterogeneity through sensitivity analysis and subgroup analysis, the potential source of heterogeneity could be found. Third, the cut-off value varied greatly across the included studies such that we could not determine the optimal NLR value for clinical use.

## Conclusion

Our study indicated that a high NLR is significantly associated with poor clinical outcomes among patients with ICH. Given that NLR is a simple and easily available biomarker, future studies should focus on exploring its application in the prognostic evaluation of patients with ICH.

## Data Availability Statement

The original contributions presented in the study are included in the article/Supplementary Material, further inquiries can be directed to the corresponding authors.

## Author Contributions

MS took responsibility for the integrity of the data and the accuracy of the data analysis. MS and X-FL contributed significantly to data analysis, data acquisition, and manuscript preparation. MS, X-FL, T-BZ, and Q-WT made critical revision of the manuscript for important intellectual content. MP guided the research and revised the manuscript. W-YZ guided the research and contributed to supervision. All authors contribute to the conception, design, analysis, and interpretation of data of the study.

## Conflict of Interest

The authors declare that the research was conducted in the absence of any commercial or financial relationships that could be construed as a potential conflict of interest.

## Publisher’s Note

All claims expressed in this article are solely those of the authors and do not necessarily represent those of their affiliated organizations, or those of the publisher, the editors and the reviewers. Any product that may be evaluated in this article, or claim that may be made by its manufacturer, is not guaranteed or endorsed by the publisher.

## Supplementary Material

Supplementary File 1 PRISMA 2009 Checklist.

Supplementary File 2 Free terms.

Supplementary File 3 Quality assessment of included studies using the Newcastle Ottawa Scale for cohort studies.

Supplementary File 4 Funnel plot of publication bias of NLR with primary outcome.
