# Supplementary material for: Prognostic Role of the Neutrophil-to-Lymphocyte Ratio in Intracerebral Hemorrhage: A Systematic Review and Meta-Analysis
Source: Front Neurosci. 2022 Mar 10;16:825859. doi: 10.3389/fnins.2022.825859 (PMC8960242; doi:10.3389/fnins.2022.825859)
Supplement: Supplementary File 2 — Free terms. [file Data_Sheet_2.PDF]

Free terms:

(1) neutrophil-to-lymphocyte ratio OR neutrophil to lymphocyte ratio OR neutrophil-lymphocyte ratio OR neutrophil/lymphocyte Ratio OR neutrophil-lymphocyte OR NLR

(2) intracerebral hemorrhage OR intracranial hemorrhage OR cerebral hemorrhage OR brain hemorrhage OR ICH
