# Supplementary material for: Prognostic Role of the Neutrophil-to-Lymphocyte Ratio in Intracerebral Hemorrhage: A Systematic Review and Meta-Analysis
Source: Front Neurosci. 2022 Mar 10;16:825859. doi: 10.3389/fnins.2022.825859 (PMC8960242; doi:10.3389/fnins.2022.825859)
Supplement: Supplementary File 3 — Quality assessment of included studies using the Newcastle Ottawa Scale for cohort studies. [file Data_Sheet_3.PDF]

supplementary file 3: Risk of bias assessment of cohort studies-the Newcastle Ottawa scale (NOS)

Studies	Selection				Comparability	Outcome			Total scores
	a	b	c	d	e	f	g	h	
Fonseca 2021	1	1	1	0	2	1	1	0	7
Menon 2021	1	1	1	0	2	1	1	1	8
Mohamed 2021	1	1	1	0	2	0	1	1	7
Radu 2021	1	1	1	0	2	1	1	1	8
Chen 2020	1	1	1	0	2	1	1	0	7
Guo 2019	1	1	1	0	2	1	1	0	7
Pektezel 2019	1	1	1	1	2	1	1	0	8
Qin 2019	1	1	1	0	2	1	1	1	8
Fei Wang 2019	1	1	1	0	2	1	1	1	8
Zhigang Wang 2019	1	1	1	0	2	1	1	1	8
Zhang 2019 A	1	1	1	0	2	1	1	0	7
Zhang 2019 B	1	1	1	0	2	1	1	1	8
Zhang 2019 C	1	1	1	0	2	0	1	1	7
Lattanzi 2018	1	1	1	0	2	1	1	1	8
Qi 2018	1	1	1	0	2	1	1	1	8
Wang 2018	1	1	1	0	2	1	1	1	8
Zhang 2018 A	1	1	1	0	2	1	1	1	8
Zhang 2018 B	1	1	1	0	2	1	1	0	7
Zhang 2018 C	1	1	1	0	2	1	1	0	7

Giede-Jeppe 2017	1	1	1	0	2	1	1	1	8
Lattanzi 2017	1	1	1	0	1	1	1	1	8
Seabra 2017	-	-	-	-	-	-	-	-	-
Sun 2017	1	1	1	1	2	1	1	1	9
Tao 2017	1	1	1	0	1	1	1	1	7
Lattanzi 2016	1	1	1	0	2	1	1	1	8
Wang 2016	1	1	1	0	2	1	1	1	8

Selection: a: Representativeness of the exposed cohort; b: Selection of the non-exposed cohort; c: Ascertainment of exposure; D: Demonstration that outcome of interest was not present at start of study

Comparability: e: Comparability of Cohorts on Basis of Design or Analysis Time to Follow-Up

Outcome: f: Assessment of outcome; g: Follow-up long enough for outcomes to occur; h: Adequacy of follow-up of cohorts
