# Supplementary figures and images for: Prognostic Role of the Neutrophil-to-Lymphocyte Ratio in Intracerebral Hemorrhage: A Systematic Review and Meta-Analysis
Source: Front Neurosci. 2022 Mar 10;16:825859. doi: 10.3389/fnins.2022.825859 (PMC8960242; doi:10.3389/fnins.2022.825859)

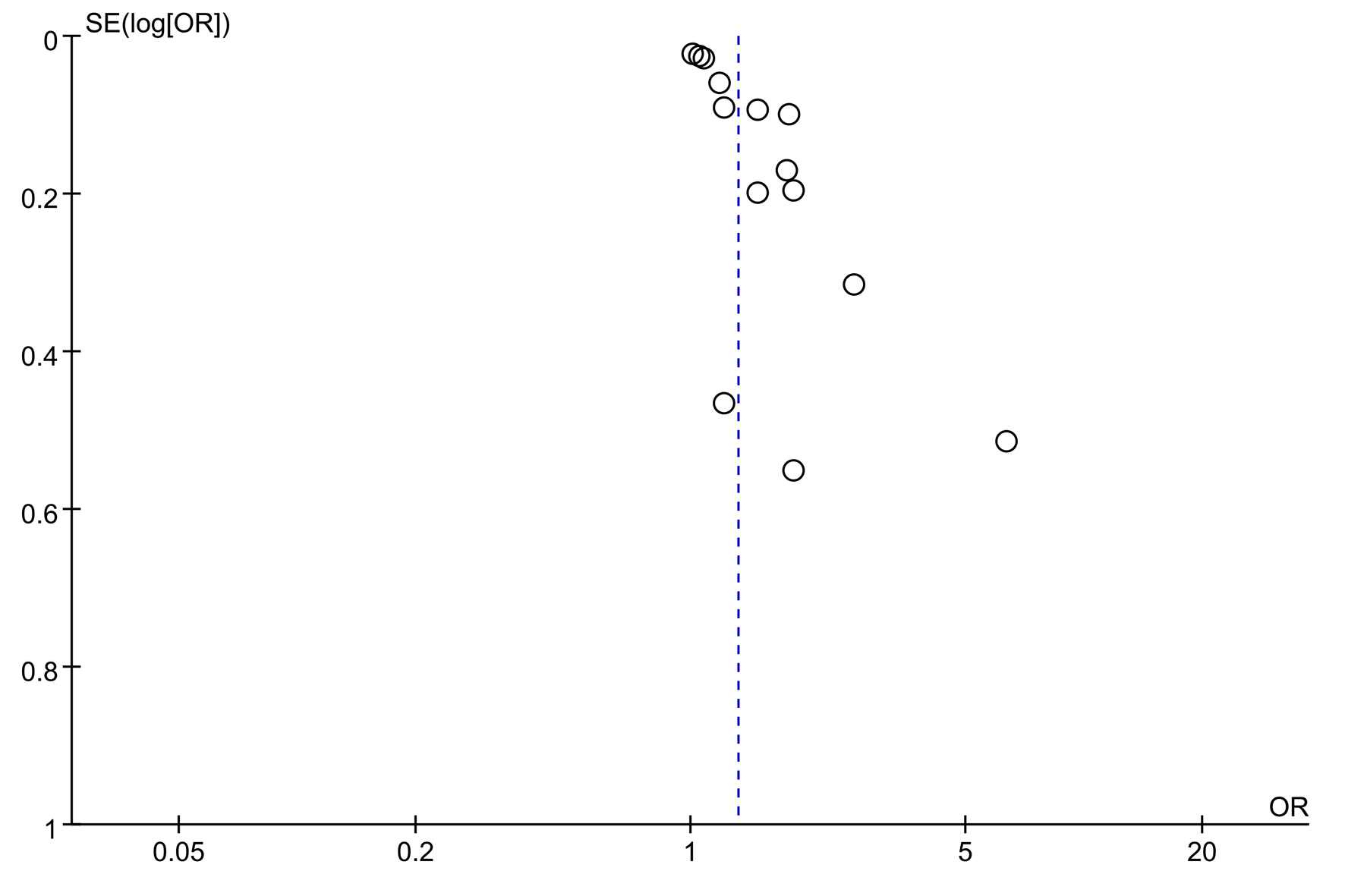

Supplement: Supplementary File 4 — Funnel plot of publication bias of NLR with primary outcome. [file Image_1.TIF]
